# Occurrence of Enteric Viruses in Surface Water and the Relationship with Changes in Season and Physical Water Quality Dynamics

**DOI:** 10.1155/2020/9062041

**Published:** 2020-07-03

**Authors:** Wasonga Michael Opere, Maingi John, Omwoyo Ombori

**Affiliations:** ^1^Department of Biochemistry, Microbiology and Biotechnology, Kenyatta University, P.O. Box 43844-00100, Nairobi, Kenya; ^2^Department of Plant Sciences, Kenyatta University, P.O. Box 43844-00100, Nairobi, Kenya

## Abstract

Environmental water quality issues have dominated global discourse and studies over the past five decades. Significant parameters of environmental water quality include changes in biological and physical parameters. Some of the biological parameters of significance include occurrence of enteric viruses. Enteric viruses can affect both human and animal's health by causing diseases such as gastrointestinal and respiratory infections. In this study, the relationship between the occurrence of enteric viruses with reference to adenoviruses and enteroviruses and the physical water quality characteristics was assessed from water samples collected from Lake Victoria (LV) in Kenya. In order to understand the dynamics of season driven enteric viruses' contamination of the lake waters, we additionally analysed seasonal behavior of the lake's catchment area in terms of rainfall effects. Physical quality parameters were measured on-site while viral analysis was carried out by molecular methods using the nested polymerase chain reaction (nPCR). From 216 samples that were analysed for viral contamination, enteric viral genomes were discovered in 18 (8.3%) of the samples. Out of half of the samples (108) collected during the rainy season, enteric viral genomes were detected in 9.26% (10) while 8 (7.41%) samples tested positive from the other half of the samples (108) collected during the dry season. There was, however, no significant correlation noted between the physical water quality characteristics and the enteric viruses' occurrence. Neither wet season nor dry season was significantly associated with the prevalence of the viruses. In Lake Victoria waters, most of the samples had an average of physical water quality parameters that were within the range accepted by the World Health Organization (WHO) for surface waters with exemption of turbidity which was above the recommended 5 NTU as recorded from some sampling sites. Continuous and long-term surveillance of the lake water to accurately monitor the contaminants and possible correlation between chemical, physical, and biological characteristics is recommended. This would be important in continuous understanding of the hydrological characteristics changes of the lake for proper management of its quality with reference to the WHO standards. A multiple varied-sampling approach in different geographical regions during different seasons is recommended to establish the geographical distribution and relatedness to seasonal distribution patterns of the viruses. The data generated from this study will be useful in providing a basis for assessment of seasonally driven fecal pollution load of the lake and enteric virus contamination for proper management of the sanitary situation around the lake.

## 1. Introduction

### 1.1. Background Information

Environmental water quality monitoring and research have been carried out around the world since 1970s [1, 2]. Key concern is the issue of pollution of environmental waters from point sources and surface runoffs which may eventually lead to significant public health related issues [3]. Indicators of environmental water quality include changes in biological, physical, and chemical attributes. The biological parameters that determine water quality include coliphage, coliform bacteria, and *Escherichia coli* [4, 5]. The presence of these biological indicators however does not necessarily correspond to the occurrence of enteric viruses such as rotavirus, adenoviruses, and enteroviruses [4, 6]. Physical quality parameters on the other hand include changes in pH, temperature, turbidity, electrical conductivity, total dissolved solids, and dissolved oxygen [7, 8]. Some of the chemical characteristics that may be of importance include concentrations of phosphorus, silicate, nitrite, alkalinity, and heavy metals such as mercury [9]. Changes in these various categories of water quality parameters may generally lead to considerable health implications and negative impact on certain important processes for livelihood in the community such as water treatment procedures [10]. Occurrence of biological parameters may sometimes be affected by the physical quality parameters [11]. For example, occurrence of pathogenic microorganisms in surface waters can be exacerbated by higher levels of turbidity which is normally due to sewage discharge [11]. Poor quality of surface waters has also been linked to other anthropogenic activities such as agriculture, construction and mining. These activities contribute to decline of the surface water quality as they expose the waters to high sedimentation during runoffs and storm water drainage during rainy seasons [12].

Surface waters quality concerns are of great significance over the world because the surface waters are one of the main sources of drinking water after treatment [13]. In Sub-Saharan Africa, one of the key surface waters whose quality has been compromised in the recent past is Lake Victoria [14]. Lake Victoria is a fresh water lake covering about 68,800 km^2^ within Kenya, Uganda, and Tanzania with the Kenyan territory covering about 4,128 km^2^ of the total surface [14]. It has a wide catchment area served by numerous rivers and streams [15]. There has been constant increase in population, urbanization, industrialization, and agricultural activities in the recent past within the lake's catchment area [16]. This has in turn led to the contamination of the lake water with different types of pollutants such as agro-based pollutants and sewage effluents from on-site sources and off-site sources via the supplying rivers and streams [17].

As a result of pollution, the quality of the lake water is compromised, thus exposing the huge surrounding population of 1,131,950 [18] that utilize its waters for economic, domestic, industrial, and agricultural purposes to health risks. The lake water quality ecosystem on the Kenyan side in particular has greatly deteriorated in the last five decades [14]. This deterioration has been exacerbated by eutrophication and acidification which have all been linked to anthropogenic impacts as a result of increase in population in the nearshore towns such as Kisumu, Homa Bay, and Mbita [19]. The population pressure and the activities required to support the expanding population have led to increased inflows of pollutants to the lake and to the waterways from the catchment area leading to poor quality [14]. A number of physical and chemical quality parameters on Lake Victoria (LV) were studied by Calamari et al. [20]. However, no comprehensive analysis has been undertaken to determine the relationship between the occurrence of biological parameters and the dynamics of physical-chemical parameters. Various studies involving biological parameters on the lake have been primarily focused on coliforms [21] and planktons [22]. Enteric viruses' studies especially on the Kenyan territory have never been reported. We analysed the situation of some physical water quality parameters and the occurrence of enteric viruses with respect to adenoviruses and enteroviruses from the Kenyan side of LV waters along Homa Bay town in Kenya.

The aims of the study were therefore as follows: (1) to determine whether the physical characteristics of water in Lake Victoria along Homa Bay town influence the occurrence of the enteric viruses; (2) to determine whether the lake water physical water quality parameters are within the World Health Organization (WHO) acceptable levels for domestic use; (3) to understand the dynamics of season driven enteric viruses' contamination of surface waters through analysis of seasonal behavior of the surface waters' catchment area in terms of variation in rainfall activities. Analyses of physical water quality dynamics in relation to the enteric viral contamination in environmental waters may be useful in proper diagnosis of environmental waters quality for proper remedial action. Six physical water quality characteristics, namely, temperature, dissolved oxygen, conductivity, pH, and turbidity, dissolved solids were analysed. For biological characteristics, enteric viruses were analysed as potential pathogens. Adenovirus (HAdV), a double-stranded DNA virus of family Adenoviridae and genus *Mastadenovirus* [23–25], and enteroviruses, (EV) a single-stranded positive sense RNA virus of family *Picornaviridae* and genus *Enterovirus* [26, 27], were considered as index viruses. Enteric viruses' infections are propagated by water mainly by drinking contaminated water or through contact with recreational water [28]. Enteric viruses are transmitted by fecal-oral route and can affect both human and animal's health by causing a myriad of diseases such as gastrointestinal and respiratory infections [29]. The relationship of enteric viruses' contamination of surface waters and changes in seasons is significant in recognition of the fact that fecal contamination of surface waters from the catchment area may be a result of collection and transportation of fecal contaminants in response to rainfall events [30]. The epidemiology of some enteric viruses has been reported to be affected by seasonal changes in temperate regions [31]. For example, enteroviruses have been reported to be at peaks during early autumn or late summer [32]. Studies have shown that viruses may survive relatively longer in lower temperature conditions [33]. However, adenoviruses, as one of the key waterborne enteric viruses, have been reported to exist in surface waters all year round independently on changes on season [34]. Effects of changes in seasonality on the distribution of such viruses in tropical regions such as in the sub-Saharan Africa has not been widely researched.

The study will give an insight into the present status of the quality of the lake water and provide a reference point for future monitoring of the water quality. The future monitoring will inform the design of appropriate management of pollution issues of the lake waters. Data regarding effects of changes in seasons on the contamination of surface waters with these viruses will be important in forming a basis for understanding of the viruses' epidemiology in the surrounding community and the potential for an outbreak. This intern would be useful in drawing of plans for control and prevention.

## 2. Materials and Methods

### 2.1. Study Site

**Figure 1:**
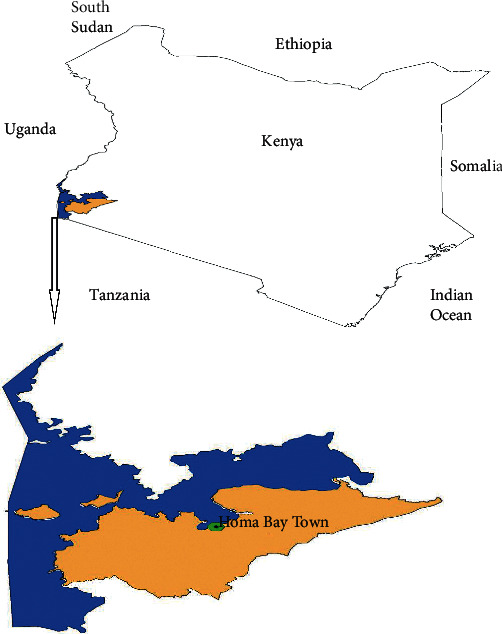
Map of Kenya showing Homa Bay County where the study was carried out.

The study site was located between longitudes 34.30°E and 34.20°E and latitudes 0.30^o^S and 0.35^o^S in Homa Bay Town, Homa Bay County in Western region of Kenya (Figure 1). The town is one of the major urban centres located nearshore on the Kenyan side of Lake Victoria. It is a significant station in relation to potential sources of contamination of the lake waters with pollutants such as agricultural, industrial, domestic, and sewage effluents. Its population has greatly increased in the last decade which currently stands at 1,131,950 [18], thus compounding the pollution pressure to the surrounding lake ecosystem. The prevailing climatic condition is tropical wet with a binomial rainfall distribution pattern [35]. The longer rainy season is normally experienced from the months of March to April while the shorter rainy season is normally experienced from the months of August to November [35]. Monthly mean temperature is normally about 11.54°C with the annual potential range for evaporation being about 1180 mm to 1322 mm [36]. The area has gently sloping terrain surrounded by hilly topography such as Asego hill. This makes the area more vulnerable to runoffs which end up into the lake during rainy seasons.

### 2.2. Sampling Sites

**Figure 2:**
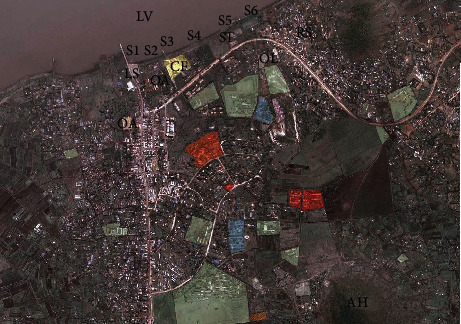
Satellite image showing the distribution of the sampling points [35]. ^*∗*^S: sampling sites, LS: landing site/Jetty, OA: open air market, CF: Capital fish factory, ST: sewage treatment plant, OL: old water treatment works, AH: Asego Hill, LV: Lake Victoria, and RS: residential sites.

Six points were selected from the study site for samples collection based on observed possible contamination impacts resulting from increased human activities of urbanization, including water transport, industrial and waste water treatment activities. The sampling sites were distributed along a strip in the nearshore of the lake in the town and were designated as sites S1 to S6 (Figure 2). S1 is located within decimals −0.52299 and 34.45524 and the nearby potential source of contamination identified was a landing site, water transport, and fishing activities within the surrounding. Site S2 is located within decimals −0.52201 and 34.45705 with potential source of contamination being a nearby open air market. The locations for the other sites were S3 −0.52115 and 34.45975, S4 −0.52079 and 34.46058, S5 −0.52001 and 34.46152, and S6 −0.51918 and 34.46404. The observed potential sources of contaminats for these sites were; S3, presence of a nearby fish processing factory (Capital fish); S4, farming activities involving animal husbandry within the surrounding; S5, presence of sewage treatment works within the vicinity; and S6, being located near a residential area and a sewage treatment plant. The physical and biological parameters of the lake waters around the selected strip are suspect going by the observed levels of human activities within the surroundings.

### 2.3. Sample Collection

**Figure 3:**
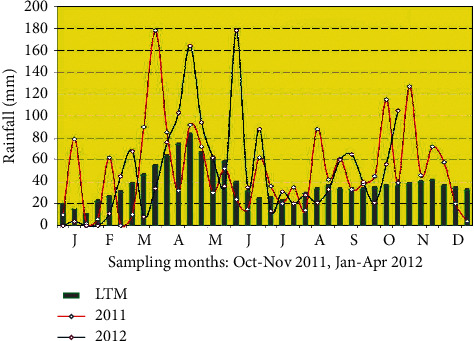
2012 rainfall distributions, Homa Bay, Nyanza. Source: FAO and KFSSG [36]. LTM: last twelve months.

Ten litres of water samples were collected using a 10-litre sterilized clean plastic container from the surface of the water at a depth of about 50 cm from each of the sampling points. The sampling was carried out for a six-month period, from October 2011 to April 2012 with the period being dichotomised as rainy or dry season. Sampling months of October 2011, November 2011, and April 2012 were rainy months, while January, February, and March 2012 were all dry months. December 2012 was not included as a sampling month so as to balance the number of sampling trips for the wet/dry season dichotomy. The number of samples per every sampling trip/month from a single site was 6, making a total of 36 samples across all the six sampling sites per trip and 216 for the entire six-month sampling period. The total sampling volume per site was 60 litres, making a total volume of 2160 litres for the entire sampling period. According to a joint report from the Food and Agriculture Organization (FAO) of the United Nations and the Kenya Food Security Steering Group (KFSSG) from 2011 to 2012 when the sampling was carried out, the highest recording of rainfall was in April 2012 with the maxim recording being about 165 mm. On the other hand, the lowest rainfall amount was recorded in January 2012 which was only about 10 mm (Figure 3).

### 2.4. Measuring of Physical Quality Parameters

The physical quality parameters were measured and recorded *in situ* in the field during sample collection according to the Standard Methods for the Examination of Water and Wastewater-the 21^st^ edition as described by the American Public Health Association [37]. Briefly, various portable water probe equipment were used to get the measurements on-site. Temperature and pH were measured by the electrode probe method using water temperature and pH meter model (Jenway, model 550). Dissolved oxygen was also analysed by an electrode method using a multimeter electrochemical analyser (Jenway, model 3405). Turbidity was analysed by the turbidimetric method using a portable turbidimeter (turbidimeter-model 2100 USA) while electrical conductivity and total dissolved solids were measured using a total dissolved solids/conductivity meter (Jenway model 4076).

### 2.5. Detection of Enteric Viral Genome from the Samples

Once the physical characteristics were recorded on-site, the samples were transported on ice to the Enteric Viruses Research Group-Institute of Primate Research Laboratory in Nairobi, Kenya, for enteric viruses' analysis where they were temporarily stored at a temperature of 4°C until processing [38]. Sample processing was done between 6 and 8 hours following collection process. Concentration of the samples and recovery of the virus were carried out using glass wool adsorption-elution technique according to the methods originally described by Vilaginès et al. [39] and subsequently modified by Wolfaardt et al. [40], Lambertini et al. [41], and Miagostovich [42]. Briefly, the process involved filtering and draining of 10 litres of the water samples through a column of a Perspex glass tube stocked with layers of positively charged oiled sodocalcic glass wool filters. The draining of the water from the glass tubes was achieved by application of a negative pressure using a vacuum pump. A few samples that had pH greater than 7.00 were normalised using 1 N HCl to adjust their pH to 7.00 before the filtration process to enhance adsorption of the viruses during the sample concentration process [43].

Being that virus particles are negatively charged, the viruses got adsorbed to the positively charged glass wool filters during the running of the sample through the perspex column [41]. The adsorbed viruses were eluted from the glass wool filters using 100 ml of glycine-beef extract buffer (GBEB), pH 9·5. One hundred millilitres of the elute was subjected to secondary concentration by washing using polyethylene glycol/sodium chloride (PEG/NaCl) according to the methods previously described by Vilaginès et al. [44]. The resultant solution of the secondary concentration was incubated overnight at 4°C and then centrifuged at 4,200 rpm for 45 min at 4 °C. The resulting pellets were resuspended in a 2 ml of sterile phosphate-buffered saline solution containing 0.15 M Na_2_HPO_4_ solution, pH 7.0, and later centrifuged at 3000 rpm for 10 min at 4°C. The supernatant was collected into a sterile container [38] and stored in separate sterile 20 ml glass tubes (Thermo Fisher Scientific) at a temperature of −70°C [45] until use for nucleic acid extractions. The virus concentrates were stored for between 2 and 12 hours before nucleic acid extraction in which 2 ml of the supernatant was later used for the process.

Nucleic acids were extracted from the 2 ml supernatant using the automated commercially available extratcion kits. The MagNA pure total nucleic acid extraction kit (Roche Diagnostics) and RNeasy minikit (QIAGEN) were used for extraction of the DNA and RNA extratcion respectively, according to the manufacturer's instructions. The DNA extracts were stored in Tris-EDTA (pH 8.0) at a temperature of −20°C until use following an elution process. The RNA extracts on the other hand were stored by freezing in RNase free water at −80°C. From 2 ml of the extracted nucleic acids solutions, nested PCR was used for amplification according to methods described by [46, 47] with minimal modifications. For the RNA genome, reverse transcription (RT) was first carried to synthesis m cDNA for enteroviruses before the PCR process. Published primers as previously described in studies of Allard et al. [48], Allard et al. [34], and Allard et al. [46] and further described by Santos et al. [49] were used for the PCR process. The PCR products were visualised using 2% agarose gel electrophoresis stained with ethidium bromide. During the handling of the samples in the laboratories, care was taken to minimise chances of cross contamination. Some of the precautions adopted include carrying out samples processing in different laboratories with different set of apparatus. Probability of amplifying contaminant DNA was reduced by treating the nucleic acid samples with uracil DNA glycosylase [50].

### 2.6. Data Analysis

Two-way analysis of variance (ANOVA) test was used to analyse comparison of the variations between the physical parameters at different seasons and the sampling sites, while Pearson correlation was used to analyse the relationship between the physical parameters and viruses' presence. SAS version 9.1(SAS Inst. INC., Car., NC) was used to carry out all the analyses in which the *p* values were considered statistically significant at *p* values <0.05.

## 3. Results

### 3.1. Occurrence of the Enteric Viruses

From a total of 216 samples that were collected for viral analysis, the enteric viral genomes were detected in 18 (8.3%) of them. Of these 18 positive samples, 8 (44.4%) were recorded from site S5, while 5 (27.78%) were from site S6 apparently suggesting higher pollution levels in the surrounding areas. Sites S1, S2, and S4 recorded 1 viral genome each, jointly accounting for only 16.67% of the total number of positive samples while site S3 had two (11.11%) positive samples (Table 1).

As regards to seasonal distribution of the viruses, the genomes were detected at least once in each of the six sites sampled during the two seasons, although no site was virus-positive in every sampling month (Table 2). Out of 108 samples collected between October and November and in April which were rainy months, 10 (9.26%) had enteric virus genomes. Similarly, out of the 108 samples collected from January to March during the dry season, 8 (7.41%) tested positive for adenovirus and enteroviruses contamination (Table 2).

As regards relationship between microbiological parameters in question (human adenoviruses and enteroviruses) and the seasons of samples collection, we observed that there was no significant difference in the number of adenovirus detection during the dry and wet season (*p* = 0.7440) (Table 3). The highest mean for the number of adenovirus genome detected although was observed during the wet season (0.06) in comparison to the dry season (0.05) (Table 3). Similarly, enteroviruses detection was not significantly different between the wet and dry seasons (*p* = 0.7010). The highest mean for enteroviruses however was observed during the wet season (0.04) while the mean detection for the dry season was (0.03).

### 3.2. Physical Quality Characteristics

The result of the six physical water quality characteristics was analysed between the wet and the dry seasons and among the six sampling sites in relation to the World Health Organization (WHO) standards for environmental waters. Generally, the average values for most of all the physical parameters were found to be in compliance with WHO acceptable levels except in few cases (Table 3). For example, the results show that most of the samples had pH average values ranging from 7.00 to 7.06 which were within the WHO acceptable levels of 6.50–8.00. Similarly, the mean temperature values varied between 25.47°C and 25.92°C for all the sampling periods as measured on-site. The results indicate that the water temperature was within the acceptable limits of ±2°C from 25.00°C. All conductivity and TDS samples were however below the thresholds recommended by the WHO at 500–5000 mS/cm and 1000 mg/l, respectively, for fresh water bodies. Higher levels of dissolved oxygen were recorded from sites S1 and S2 which are characterized by slightly intense anthropogenic activities. Average DO values at these two sites were 9.55 mg/l and S2, 9.46 mg/l, which is above the WHO recommended values of 8–9 mg/l. On the other hand, lower DO values than the recommended range were recorded from site S6, with mean DO levels from the site being 7.98 mg/l. It is noteworthy that this site is located close to a residential place and not so distant from a sewage treatment plant; the possibility of its samples being highly contaminated is real from possible domestic discharge and sewage leaks. Majority of the samples nonetheless recorded DO levels which were within the WHO recommended range. As for turbidity values, all the average values in the various sites and seasons were found to be above the recommended values in comparison to the WHO guidlines for environmental waters of 5 NTU (Table 3).

Analysis of these parameters between the two seasons and among the different sampling sites revealed that there were generally significant differences with the probability values being *p* ≤ 0.5. For instance, seasonal analysis of electrical conductivity indicates that there was a significant difference (*p* ≤ 0.5, *p* = 0.0001) during the dry and wet seasons. Significant difference was also noted in changes on values between the sites (*p* = 0.0001) (Table 3). In terms of recorded values between the wet and the dry seasons, the highest mean conductivity was observed during the dry season (70.73 *μ*S/cm) while the lowest mean conductivity was observed in dry season (0.28 *μ*S/cm) (Table 3). On the other hand, conductivity values per site indicate that the highest mean conductivity was observed in site S2 (60.02 *μ*S/cm) while the lowest mean conductivity was observed in site S5 at 3.91 *μ*S/cm (Table 3). Similarly, difference in turbidity levels was strongly significant between the seasons as well as the sites (*p* = 0.0001). The highest mean value in terms of season was 19.73 NTU during the wet season while in terms of sites, it was in site S5, being 21.50 NTU.

We evaluated the relationship of the occurrence of the two microbial parameters (HAdV and EV) in the water samples and the six physical water quality dynamics (temperature, electrical conductivity, total dissolved solids, dissolved oxygen, pH, and turbidity). The occurrence of the viruses did not show any significant relationship with any of the physical parameters (Table 4). However, there were significant associations between the physical parameters themselves. For example, there was a strong positive correlation between TDS and EC (*r* = 0.331, *p* = 0.0001) and a strong negative correlation between turbidity and EC *r* = −0.542, *p* = 0.0001).

## 4. Discussion

### 4.1. Occurrence of the Viruses from the Water Samples

Enteric viruses such as HAdV and EVs have been discovered in many of surface waters around the world [51–53]. In this study, the genomes of these viruses were discovered in 8.3 % of the 216 samples analysed. This corroborates with other findings that have been reported around the world from different studies as regards the occurrence of enteric viruses in surface waters [54]. Based on the analysis of seasonal viral occurrences, we observed that virus detections varied through the sampling months that included both the wet and the dry seasons from individual sampling sites. Rainfall activities may lead to increase in the level of contamination of the surface waters by enteric viruses as a result of occasional discharges from surface runoffs [30]. During the sampling period, it was observed that there was higher rainfall activity in the month of October 2011, and thus, the lake received a lot of runoff water in this first month of sampling. However, the analysis show that there was neither a significant higher detection nor increase in the number of viruses detected in the following month of November which was also a wet season. In general, the detection of the viruses in this study did not show any significant change during the flooding heavy rainfall months of October 2011, November 2011, and April 2012 and the three dry months. These results nonetheless are consistent with a past study reported in Sub-Saharan Africa by Ayukekbong et al. [31] in Cameroon and elsewhere [55–57]. Other studies although have revealed seasonal effects on detection of other types of enteric virus in certain environmental waters. For example, human caliciviruses have been found to be more prevalent during the colder months of the year [58]. Adenoviruses and enteroviruses have also been reported to show relatively little change in concentration with changes in seasonal trends throughout the year [59].

Studies have found that virus can persist suspended in environmental waters for several days at both very low and high temperatures [5]. It has been reported from past studies that viral persistence in the environmental waters increases with decrease in temperature [60]. This means that viruses are likely to persist more if the lake is cooler than when warm [61]. According to the results, there was no significant difference in variation in temperature recorded during both the wet and the dry seasons and this could be one of the reasons as to why there was no significant difference in the detection of viruses in both dry and rainy seasons. Viruses have been isolated throughout the year with slight increase in concentration during rainy seasons although not always [60, 62]. Despite the fact that there was no significant change in detection during wet seasons, it can be assumed that viruses may have been transported to the sampling sites later after getting desorbed from subsurface sediments after infiltration of rainfall and hence can be detected at a later date [57].

Studies have shown that there are chances of increase in ease of transportation of viruses with increase in water flow and this may increase chances of viral detection [63]. However, few viruses were detected during increased water flow period in the wet months and a possible explanation for this could be the dilution effect of the flooding activities [56]. During dry seasons, less water flow happens but contamination of the lake water by the viruses can still occur through aquifer pathways [64]. This could explain the near similar detection of the viruses during the dry season. Virus detection during dry season however is not affected by the dilution effect due to less water flow [65]. Contamination during the dry season can be through the ground flow probably exacerbated by the positive surface waters which is in contrast with the wet season when contamination is likely to be through water flow in the upper part of the soil [29].

Higher detection of viruses from some of the sites such as sites S5 and S6 could be an indication to high level of contamination or discharge of pollutants around the sites. This could be attributed to intense human activities from pressure of urbanization leading to sewage and industrial and agricultural discharges to the lake. Lower detection from sites S1, S2, and S3 could as well be associated with factors that may have hindered PCR detection of the viruses. Certain conditions and chemicals have been reported to limit PCR amplification [66]. Some of these factors include waters with high concentration of humus, phenols, and heavy metals [67].

### 4.2. Physical Quality Characteristics

Most of the samples from all the sites recorded physical water quality characteristics that were within the recommended range of values according to the World Health Organization except for turbidity which was higher in most of the samples. The higher levels of turbidity however could be attributed to agricultural activities from the surrounding community especially during the rainy seasons as a result of runoff carrying silt [8]. The mean average temperature of the samples was 25°C with a range of 24°C to 26°C which falls within the recommended temperature ranges for inland waters and suitable for aquatic life which does not thrive well when the temperature changes by a ±2°C [68, 69]. There were high concentrations of dissolved oxygen recorded in some cases although a larger percentage fell within the WHO recommended range of 8-9 mg/l for surface water. A DO below 5 mg/l signifies a highly polluted water and not suitable for aquatic life and may act as an indicator for poor water quality [70]. There was a variation of results of DO with a mean range of 7.98–10.40 5 mg/l recorded across the two seasons indicating presence of limited organic waste in the lake. Samples from site S6 recorded slightly lower value than the recommended range, with mean DO levels of 7.98 mg/l. This lower value could be attributed to increased discharge of untreated industrial and urban wastewater [71].

### 4.3. Analysis of the Relationship between Physical Parameters and Biological Parameters

Relationship between physical water quality parameters and viral contamination in surface waters has been reported in previous studies [72]. Positive correlation has been reported between some physical parameters such as turbidly and PH with enteric viruses' presence in surface waters [73]. Higher turbidity in particular has been reported to contribute to difficulty in executing various water purification processes such as flocculation and filtration, thereby increasing the cost of water treatment and consequently may lead to increased chances of fecal pollution and enteric viruses' contamination [62]. We conducted a longitudinal monitoring for 6 months, in which two microbiological parameters (adenoviruses and enteroviruses) situation and the relationship with physical quality parameters (pH, temperature, turbidity, dissolved oxygen, electrical conductivity, and total dissolved solids) were assessed. Generally, there was no significant correlation observed between the microbiological and physical parameters. However, among the 6 physical parameters evaluated and correlated with the microbiological parameters, turbidity was the only one found to be significantly above the recommended level by the WHO. However, this does not seem to have had an effect on its correlation with the biological parameters. This result is consistent with previous studies where no correlation was recorded between the occurrence of viruses and the changes of physical quality parameters. For example, Lee et al. [74] reviewed and documented absence of correlation between biological and physical parameters. The occurrence of viruses in water for example did not correlate with physical factors such as pH and turbidity. Similar reports by Lee et al. [74] have reported lack of correlation between enteroviruses presence in surface waters with physical parameters such as turbidity and temperature of the waters.

## 5. Conclusions

Most of the samples had an average of the physical water quality parameters that were within the range accepted by the WHO. However, the detection of turbidity above the levels recommended by the WHO and DO concentrations in some cases at levels below the recommended threshold is a justification that the level of pollution of the lake water is high and therefore the lake environment should be subjected to continuous monitoring for proper management of pollution challenges. Adenoviruses and enteroviruses occurrence in LV waters are relatively constant throughout the year without significant variations in their profile with changes in season. Changes in season dynamics may therefore be considered to be an unreliable factor for prediction of viral contamination peaks in the lake.

In addition to these findings, the physical and microbiological parameters were found not to be significantly correlated; the occurrence of the viruses was not affected by changes in the physical parameters. We recommend a more detailed continuous long-term surveillance of the lake waters to accurately monitor the contaminants and possible correlation between chemical parameters and other physical characteristics not covered in this study such as light penetration and the virus occurrence. There was no significant influence of season in detection of the viruses as per the present study; however, a multisampling approach in different regions and during different seasons is recommended to establish the viruses' geographical distribution and relatedness to seasonal distribution patterns.

## Figures and Tables

**Table 1:** Summary of the bivariate analysis for the viral contamination by site.

Sampling sites	No. of samples (*n*)	Contamination			
		Virus absent		Virus present	
		Frequency	(%)	Frequency	(%)
S1	36	35	97.22	1	2.78
S2	36	35	97.22	1	2.78
S3	36	34	94.44	2	5.56
S4	36	35	97.22	1	2.78
S5	36	28	77.78	8	22.22
S6	36	31	86.11	5	13.89

**Table 2:** Summary of virus detection from each site during the wet and the dry seasons.

Month	Season	Enteric virus	L1	L2	L3	L4	L5	L6
October 2011	Wet	Enterovirus	0	0	0	0	0	0
		Adenovirus	0	0	0	0	1/11	0

November 2011	Wet	Enterovirus	0	0	0	0	2/7	0
		Adenovirus	1/11	0	0	0	2/11	0

January 2012	Dry	Enterovirus	0	0	0	0	1/7	0
		Adenovirus	0	0	2/11	0	0	0

February 2012	Dry	Enterovirus	0	0	0	1/7	0	0
		Adenovirus	0	0	0	0	0	2/11

March 2012	Dry	Enterovirus	0	1/7	0	0	0	0
		Adenovirus	0	0	0	0	0	1/11

April 2012	Wet	Enterovirus	0	0	0	0	0	2/7
		Adenovirus	0	0	0	0	2/11	0

The numerator represents the number of viruses detected in a given month, while the denominator represents the total number of the specific virus detected during the whole period. Zeroes represent nondetection.

**Table 3:** Two-way analysis of variance with post hoc analysis using Tukey's HSD on physical parameters and viruses present.

Treatment	pH	Temp. (°C)	EC (*μ*S/cm)	TDS (mg/l)	DO (mg/l)	Turbidity (NTU)	HAdV detected	EV detected
Season								
Dry	7.00 ± 0.00a^*∗*^	25.90 ± 0.03a	70.73 ± 3.89a	50.14 ± 3.24a	8.81 ± 0.12a	9.98 ± 0.25a	0.05 ± 0.02a	0.03 ± 0.02a
Wet	7.03 ± 0.011072b	25.56 ± 0.07a	0.28 ± 0.05b	47.56 ± 2.08b	8.58 ± 0.11b	19.73 ± 0.85b	0.056 ± 0.02a	0.04 ± 0.02a

Site								
S1	7.00 ± 0.0b	25.47 ± 0.13c	56.97 ± 11.40a	87.17 ± 6.13a	9.55 ± 0.20a	6.06 ± 0.25e	0.03 ± 0.03a	0.03 ± 0.03a
S2	7.00 ± 0.00b	25.78 ± 0.07abc	60.02 ± 10.14a	30.58 ± 1.66c	9.46 ± 0.24a	16.00 ± 1.05c	0.00 ± 0.00b	0.03 ± 0.03a
S3	7.00 ± 0.00b	25.83 ± 0.06ba	34.63 ± 5.90b	50.72 ± 1.58b	8.46 ± 0.17bc	11.00 ± 0.40d	0.06 ± 0.04a	0.00 ± 0.00a
S4	7.00 ± 0.00b	25.53 ± 0.13bc	20.62 ± 3.38c	25.47 ± 0.78d	8.63 ± 0.15b	15.50 ± 1.11c	0.00 ± 0.00b	0.00 ± 0.00a
S5	7.06 ± 0.03a	25.83 ± 0.06ab	3.91 ± 0.00c	50.00 ± 3.66b	8.08 ± 0.11bc	21.50 ± 1.56a	0.14 ± 0.06a	0.08 ± 0.05a
S6	7.03 ± 0.02ab	25.92 ± 0.05a	4.25 ± 0.00c	49.17 ± 3.52b	7.98 ± 0.10c	19.08 ± 1.34b	0.08 ± 0.05a	0.06 ± 0.04a
WHO standards	6.5–8.5	25°C ± 2	500–5000	500–1000	8–9	*<*5		
*P* values								

Season	0.0090	<0.0001	<0.0001	0.0028	0.0447	<.0001	0.7440	0.7010
Site	0.0077	0.0002	<0.0001	<0.0001	0.0447	<.0001	0.0373	0.3046

^*∗*^Values followed by the same letter along the column are not significantly different based on Tukey's HSD at *p* ≤ 0.5. EC: electrical conductivity, TDS: total dissolved solids, and DO: dissolved oxygen.

**Table 4:** Correlation of physical parameters and viruses present.

	pH	Temp. (°C)	EC	TDS	DO	Turbidity	HAdV detection	EV detection
Temp. (°C)	^*∗*^0.032							
	0.636							
EC	−0.131	0.267						
	0.055	0.0001						
TDS	0.125	0.147	0.331					
	0.067	0.031	0.0001					
DO	−0.108	0.012	0.245	0.214				
	0.115	0.850	0.0001	0.002				
Turbidity	0.247	0.011	−0.542	−0.132	−0.157			
	0.0001	0.874	0.0001	0.053	0.021			
HAdV detection	0.089	0.114	−0.062	0.045	−0.083	0.108		
	0.193	0.096	0.366	0.508	0.222	0.113		
EV detection	0.128	0.090	−0.027	0.119	−0.068	0.087	−0.042	
	0.060	0.188	0.693	0.082	0.317	0.202	0.535	

^*∗*^Cell contents: Pearson correlation (*r*); *p* value at 0.05 probability level. Temp: temperature, EC: electrical conductivity, TDS: total dissolved solids, DO: dissolved oxygen, HAdV: human adenovirus, and EV: enteroviruses.

## Data Availability

The metadata used to support the findings of this study have been deposited in the Kenyatta University Institutional repository at http://ir-library.ku.ac.ke/handle/123456789/19905.
